# Prenatal Management Strategy for Immune-Associated Congenital Heart Block in Fetuses

**DOI:** 10.3389/fcvm.2021.644122

**Published:** 2021-04-28

**Authors:** Hongyu Liao, Changqing Tang, Lina Qiao, Kaiyu Zhou, Yimin Hua, Chuan Wang, Yifei Li

**Affiliations:** Key Laboratory of Birth Defects and Related Diseases of Women and Children of Ministry of Education (MOE), Department of Pediatrics, West China Second University Hospital, Sichuan University, Chengdu, China

**Keywords:** fetal immune-associated heart block, prenatal diagnosis, prenatal management, transplacental drug administration, outcome

## Abstract

Fetal congenital heart block (CHB) is the most commonly observed type of fetal bradycardia, and is potentially life-threatening. More than 50% of cases of bradycardia are associated with maternal autoimmunity, and these are collectively termed immune-associated bradycardia. Several methods have been used to achieve reliable prenatal diagnoses of CHB. Emerging data and opinions on pathogenesis, prenatal diagnosis, fetal intervention, and the prognosis of fetal immune-associated CHB provide clues for generating a practical protocol for clinical management. The prognosis of fetal immune-associated bradycardia is based on the severity of heart blocks. Morbidity and mortality can occur in severe cases, thus hieratical management is essential in such cases. In this review, we mainly focus on optimal strategies pertaining to autoimmune antibodies related to CHB, although the approaches for managing autoimmune-mediated CHB are still controversial, particularly with regard to whether fetuses benefit from transplacental medication administration. To date there is still no accessible clinical strategy for autoimmune-mediated CHB. This review first discusses integrated prenatal management strategies for the condition. It then provides some advice for clinicians involved in management of fetal cardiovascular disorder.

## Highlights

- This review first summarizes approaches for managing autoimmune-mediated congenital heart block based on the advantages and disadvantages of medications administration.
- Previous studies provide evidence that supports positive treatment for first-degree and second-degree atrioventricular blocks.
- Dexamethasone and hydroxychloroquine have not demonstrated any advantages with respect to reversing first-degree fetal atrioventricular block, but it is still recommended to administer medication for first-degree atrioventricular block due to therapeutic effects of dexamethasone and hydroxychloroquine to prevent progression of heart block.
- Transplacental administration of dexamethasone, intravenous immunoglobulin, and hydroxychloroquine is not considered viable for reversing third-degree atrioventricular blocks or benefiting fetal prognosis.
- Treatment to maintain heart function via digoxin and a β-sympathomimetic benefit delivery outcomes, but some patients born under such circumstances require pacemaker implantation soon after birth.
- Anti-Ro/SSA and La/SSB antibodies contribute to the most common causes of fetal immune congenital heart block.

## Introduction

Congenital heart block (CHB) is one of the most commonly observed types of fetal bradycardia, and it entails potentially life-threatening problems for the fetus, and can result in fetal developmental delay and even intrauterine death (1). The general fetal mortality rate associated with CHB is 19%, and in ~70% of cases a pacemaker is implanted after birth. CHB is usually diagnosed between 18 and 24 gestational weeks (GWs) during pregnancy via fetal echocardiography techniques, but in some cases it has reportedly been identified at 16 GWs. In some late onset cases it has not been detected until 29 GWs. Fetal CHB is considered a dynamic condition, because some patients with second-degree or complete atrioventricular block (AVB) exhibit a normal atrioventricular interval or first-degree AVB in initial screening during pregnancy. Fetuses with severe conditions usually exhibit a significant reduction in ventricular rate, between 50 and 70 beats per minute (2), and in such cases there is a higher likelihood of fetal heart failure, edema, premature delivery, abortion, or intrauterine death.

Current evidence indicates that CHB is almost universally associated with transplacental transfer of Ro and La antibodies, which causes cardiac conduction tissue damage that can occur as early as 11 GWs (3). To date however, the underlying molecular mechanisms of CHB pathogenesis have not been fully elucidated. Approaches to CHB management are still controversial, and decisions are always made based on the advantages and disadvantages of administration. Emerging evidence suggests that early diagnosis and proper treatment can improve the prognosis and survival rate of affected fetuses, and some common opinions pertaining to the prenatal management of CHB have emerged (1, 4).

Herein we present a review of the autoimmune antibodies related to CHB. The review is an attempt to emphasize the practical data and opinions on the pathogenesis and prenatal diagnosis of fetal immune-associated CHB, particularly with respect to fetal intervention and prognosis.

## Pathogenesis

Autoimmune-associated CHB occurs in 2–5% of pregnancies in anti-Ro/SSA and anti-La/SSB antibody-positive women. It has a recurrence rate of 12–25% in a subsequent pregnancy after the birth of a baby with neonatal lupus (5–7). Anti-Ro/SSA and anti-La/SSB are both antinuclear antibodies that can cross the placenta via FcγRn, and induce autoimmunity. Studies have confirmed that autoimmune-associated CHBs often co-occur in a variety of maternal autoimmune disorders, including Sjogren's syndrome, systemic lupus erythematosus, rheumatoid arthritis, and undifferentiated connective tissue disease (8–10).

Ro/SSA antigen is a ribonucleoprotein complex. It is composed of Ro52 and Ro60 peptides. Early demonstration of the presence of immunoglobulin deposition in the heart samples of human fetuses that died from CHB provided the first evidence of an association between maternal anti-Ro antibodies and fetal CHB initiation (11, 12). It is now known that there is a strong association between anti-Ro/SSAs and the occurrence of CHB (13). A mouse model with Ro60 and La antibodies exhibits first-degree AVB in offspring (14).

In the fetal heart, Ro52, Ro60, and La antigens are located in the nuclei of cardiomyocytes and cardiac conduction cells. Ro52 is involved in the regulation of interferon regulatory factor-mediated immune responses (15–17), whereas Ro60 is thought to play a role in RNA regulation (18). Ambrosi and Wahren-Herlenius (19) proposed a biphasic model incorporating the apoptosis hypothesis and the cross-reactivity hypothesis to explain how autoantibodies from the maternal circulation lead to fetal immune-associated CHB. The apoptosis hypothesis suggests that antibodies may bind to the surfaces of cells undergoing programmed apoptosis after entering the fetal cycle, resulting in apoptosis fragmentation. These apoptotic fragments are phagocytized by macrophages via podsolization, triggering a series of proinflammatory and fibrotic substrates downstream. The “waterfall effect” of cell factors involves the recruitment of white blood cells and complement, induces an immune inflammatory response and a fibrotic response, and ultimately leads to cardiac conductive cell damage, resulting in fetal immune-associated CHB. Single cell RNA-sequencing has been used to analyzed the cellular characteristics of cardiomyocytes and non-cardiomyocytes in hearts of fetal CHB. Increased and heterogeneous interferon responses were identified in varied cell types of the CHB heart, and gene expression enriched in extracellular matrix organization and fibrosis formation (20). The cross-reactivity hypothesis suggests that fetal CHB is primarily associated with maladjustment of calcium channels. In previous studies IgG extracted from the sera of mothers whose fetuses suffered immune CHB could inhibit L-type and T-type calcium channels of ventricular cardiomyocytes, sinoatrial node cells, and atrioventricular node cells, affecting electrical activity (21). Overexpression of L-type calcium channels in mice can reportedly partially ameliorate CHB due to anti-Ro/La antibody exposure (22). Abnormal electrical activity of the atrioventricular node in the heart caused by a cross-reaction would lead to chronic inflammation and fibrogenesis, which is thought to be one of the primary mechanisms of CHB induction. Thus, it has been suggested that the pathophysiological process of fetal CHB is aggressive and irreversible, due to localized cardiomyocyte and conduction cell damage and fibrosis formation (19).

In some recent studies CHB only developed in a small proportion of anti-Ro-positive pregnancies, with various outcomes (4). Given the low penetrance, it appears reasonable that other risk factors may affect the autoimmune damage to the fetal heart conduction system, such as genetic background variants and placental dysfunction. The protein sequences of the arginine 308 allele in the tumor necrosis factor alpha promoter and the leucine 10 allele in transforming growth factor beta in fetuses were significantly associated with the prevalence of immune CHB (23). Clancy et al. (24) demonstrated that 17 human leukocyte antigen gene mutation sites were significantly correlated with cardiac damage in children with neonatal lupus erythematosus. Several polymorphic sites of MHC-I polypeptide-related sequence B and tumor necrosis factor alpha are reportedly significantly associated with CHB (24). Maternal age, thyroid function, and gestational season are also evidently associated with the occurrence of immune CHB (25).

## Prenatal Diagnosis

Several methods have been used to definitively diagnose fetal CHB, including fetal echocardiography, fetal electrocardiography, and fetal magnetocardiography. In theory, fetal electrocardiography can accurately reflect fetal cardiac electrical activity, but is not commonly used in clinical practice because it is compromised by fetal movement which can lead to loss of signal, and it is also difficult to distinguish fetal signals from maternal signals. Fetal magnetocardiography is another non-invasive technique that can detect cardiac electrophysiological activity, but its application is limited by the need for expensive and specialized equipment, thus it is only available in a few regions. But magnetocardiography, likely to be a useful future diagnostic tool. In recent years, magnetocardiography has become one of the new research hotspots in the field of fetal arrhythmia because of its non-contact, high accuracy. Fetal sebaceous glands and maternal factors such as cervical insulation often affect the accuracy of fECG and fetal echocardiography results. Because magnetic conduction is not limited, fMCG can penetrate the cervix and sebaceous glands to separate the fetal magnetic signals from the maternal magnetic signals, and more directly reflect the fetal magnetic conditions. Multiple studies had shown that fMCG can provide a long-term rhythm analysis, show the mechanism of abnormal rhythm, measure the ventricular repolarization time, assess the risk of fetal sudden death and help clinicians choose more appropriate drugs (26–29).

Fetal echocardiography is currently the most common and effective approach used to identify fetal CHB. It assesses the mechanical consequences of arrhythmia rather than directly detecting the conduction signaling itself, and this can be achieved via M-mode and/or the Doppler method. M-mode echocardiography can simultaneously measure atrial motion (a wave) and ventricular motion (v wave) through the sampling line to determine the time sequence of atrial and ventricular motion, and atrioventricular conduction can then be inferred. Due to the limitations of atrioventricular contraction, peak value, and the variable position of the fetus during pregnancy however, it is still difficult to accurately measure the sampling line perpendicular to the atrioventricular node (30). Pulsed wave Doppler echocardiography (PD) can measure simultaneous flow across the mitral and aortic valve, and in cases of complete CHB it can demonstrate dissociation of atrial inflow and ventricular outflow (30). This can also be evaluated with simultaneous Doppler flows in the superior vena cava and aorta, as well as in the pulmonary vein and pulmonary artery (31). In patients with first-degree and second-degree heart block, measurements of differences between the onset of atrial and ventricular waveforms are used to calculate mechanical atrioventricular intervals (32). Tissue Doppler imaging (TDI) can directly record the mechanical activity of the atria and ventricles during the cardiac cycle, facilitating more accurate measurement of cardiac intervals (31–33).

The above-described fetal echocardiography techniques are sufficient for distinguishing and interpreting certain types of fetal CHB, particularly second-degree and third-degree AVB. in terms of the diagnosis of fetal first-degree AVB however, differences between PD and TDI need to be further balanced because the different methods to determine fetal atrioventricular intervals and the reference data of atrioventricular intervals vary significantly between the two approaches (30). The measurement of atrioventricular intervals using PD can be performed in two ways; from the mitral valve to the aorta (MV-Ao), and from the beginning of the retrograde venous a wave in the superior vena cava to the beginning of the aortic ejection wave (SVC-Ao) (31), which is influenced by fetal position and orientation. The TDI-derived atrioventricular interval is measured from the atrial contraction to the isovolumeric contraction (Aa-IV) or from the atrial contraction to the ventricular systole (Aa-Sa) at the right ventricular free wall (32). It has been reported that the atrioventricular interval of PD-derived SVC-Ao is shorter than that of MV-Ao however, and the TDI-derived Aa-IV is likely to be shorter than Aa-Sa (31, 32), and Aa-IV measurement underestimates the PR interval (33). Nii et al. concluded that TDI-derived Aa-IV tracks the PR interval more closely than PD, and may be a more accurate ultrasound method for assessing fetal atrioventricular conduction (32). In another study, the difference between TDI and PD was less evident, and atrioventricular times measured via TDI were longer, and it was suggested that applying the proposed cut-off value (> 150 ms) would lead to over-diagnosis and over-treatment of many fetuses at risk (33). It has also been reported that the atrioventricular interval is positively correlated with gestational age (32). A fixed cut-off independent of gestational age and method of measurement is not useful for diagnosing fetal first-degree AVB (33). Therefore, the prenatally accurate diagnosis of first-degree AVB remains poorly defined and it is crucial to diagnose fetal AVB using an adequate screening strategy with appropriate reference ranges.

## Prenatal Management

After maternal serum autoantibodies cross the placenta into the fetal circulation, which begins at 11 GWs, an immune inflammatory response can cause fetal CHB. Thus, in pregnant women whose serum antibody test is positive, prenatal management has important clinical implications with regard to fetal prognosis. The objective of prenatal management is to prevent exacerbation of the early cardiac inflammatory response, and improve the survival rate and prognosis, as well as promoting the safety of pregnant women.

Accurate prenatal diagnosis is the cornerstone of prenatal monitoring and therapy. Pregnant women with clinical and subclinical autoimmune diseases should be referred for fetal cardiac morphology screening and heart function evaluation. If fetal CHB is suspected, the fetal cardiac conduction interval (AV interval) should be closely monitored (34). In a recent study (35) fetal atrioventricular intervals were a poor predictor of CHB progression, but CHB surveillance does facilitate the detection of fetuses with second-degree and third-degree AVB shortly after its development, potentially resulting in timely treatment initiation and a better outcome. As stated above, fetal magnetocardiography is a very useful tool and can provide much detailed information.

The management of CHB relies on interpretation of the atrioventricular interval. The normal range of the fetal atrioventricular interval varies depending on GW and fetal heart rate. It has previously been proposed that if the fetal atrioventricular interval is > 140 ms more frequent fetal echocardiography should be scheduled, and if it is > 150 ms first-degree AVB should be diagnosed (34).

### First-Degree AVB

Balancing the benefits and adverse effects of treatment for fetal first-degree AVB is important because little is known about the natural progression of the condition. As indicated by the cases summarized in Table 1 (35–46), there is still limited evidence on the effects of fluorinated steroids such as dexamethasone on the course of first-degree immune-mediated AVB *in utero*. Progression to third-degree AVB occurred in 2/25 (8%) treated fetuses in whom postnatal pacemakers were not implanted (36). For unclear reasons however, a permanent, rate-modulated, single-chamber, ventricular endocardial pacemaker was implanted a few days after birth in an infant who had undergone sinus conversion prior to delivery. Interestingly, none of the untreated fetuses developed high-degree AVB (36). Persistent first-degree AVB was present in 6/25 (24%) treated fetuses and 10/27 (37%) untreated fetuses before birth. Persistent first-degree AVB was detected in 4/25 (16%) infants who had undergone treatment *in utero* and 3/27 (11%) infants who were untreated. Due to the very small number of cases and the consequent lack of statistical power, there are no statistically significant associations between dexamethasone therapy and any of the observed outcomes. A few studies have investigated the administration of hydroxychloroquine (HCQ), but as yet it is not yet possible to conclude whether HCQ has therapeutic or preventative effects on heart block regression (40, 41, 46).

**Table 1:** Literature summery about the initial diagnosis of fetal first-degree autoimmune-associated congenital heart block.

**References**	**Cases n**.	**Diagnosed methods**	**GWs**	**Mother's disease**	**Mother's positive autoantibody (titer)**	**Fetal AV intervals (ms) [z-scores]**	**Time of first therapy after diagnosis**	**Therapy medicines (dose)**	**Treated duration**	**Potential adverse effects of steroids**	**Prenatal results before birth**	**Postnatal therapy**	**Follow-up time**	**Follow-up results**
Vesel et al. (36)	Case 1	PD	25	SS	Anti-SSA/Ro	185	0	Dex. (4 mg/day)	To 29 w	Severe oligohydra-mnio	1	Ventricular endocardial pacemaker	4 m	1
Friedman et al. (34, 37)	Case 2	PD MV-Ao	20	SLE/UAS/asym.?	anti-SSA/Ro (Mean 16128)	165	0	Dex. (4 mg/ day)	Until delivery	–	1	–	1 y	1
	Case 3	PD MV-Ao	22	SLE/UAS/asym.?	Anti-SSA/Ro (Mean 16128)	160	0	Dex. (4 mg/day)	To 26w	Oligohydra-mnio	2	–	1 y	1
Rein et al. (38)	Case 4	FKCG	21	SLE	anti-SSA/Ro anti-SSB/La	149 [≥2]	0	Dex. (4 mg/day)	Until delivery	–	1	Pre. (0.1 mg/kg for 6 w)	1–6 y	1
	Case 5	FKCG	25	SS	Anti-SSA/Ro Anti-SSB/La	126 [≥2]	0	Dex. (4 mg/day)	Until delivery	–	1	Pre. (0.1 mg/kg for 6 w)	1–6 y	1
	Case 6	FKCG	26	SLE	Anti-SSA/Ro Anti-SSB/La	110 [≥2]	0	Dex. (4 mg/day)	Until delivery	–	1	Pre. (0.1 mg/kg for 6 w)	1–6 y	1
	Case 7	FKCG	34	SLE	Anti-SSA/Ro Anti-SSB/La	134 [≥2]	0	Bet.+Dex. (4 mg/day)	Until delivery	–	1	Pre. (0.1 mg/kg for 6 w)	1–6 y	1
	Case 8-A	FKCG	32	MCTD	Anti-SSA/Ro Anti-SSB/La	115 [≥2]	0	Dex. (4 mg/day)	Until delivery	–	1	Pre. (0.1 mg/kg for 6 w)	1–6 y	1
	Case 8-B		33			115 [≥2]				–	1	Pre. (0.1 mg/kg for 6 w)	1–6 y	1
Jaeggi et al. (39)	Case 9	Fetal echo	23	–	Anti-SSA/Ro (>100 U/ml)	250 [+14]	0	Steroids +IVIG	–	–	2	–	–	5
Izmirly et al. (40)	Case 10-11	–	–	SLE	Anti-SSA/Ro Anti-SSB/La	–	–	Dex.	–	–	1	–	–	1
Tunks et al. (41)	Case 12	PD MV-Ao	22	SLE	Anti-SSA/Ro (mean 443) Anti-SSB/La (mean 334.7) Anti-Smith (mean 40.3) Anti-RNP (mean 57)	–	0	Dex. (4 mg/day)	–	–	3 (2 days later)	–	–	3
	Case 13	PD MV-Ao	–	–		150–160?	0	Dex.	Until delivery	–	1	–	–	1
	Case 14		–	–		150–160?	0	Dex.	Until delivery	–	1	–	–	1
	Case 15		–	–		150–160?	0	Dex.	Until delivery	–	2	–	–	2
	Case 16		19+	SS, Hypothy-roidism		–	0	Steroids+ IVIG+dex.	Until delivery	–	2	–	–	2
Cuneo et al. (42)	Case 17	PD MV-Ao	19+	–	Anti-SSA/Ro	–	0	Dex.	-	–	1	–	–	1
Sonesson et al. (35)	Case 18	PD MV-Ao +SVC-Ao	23	SS	Anti-Ro52	165 [6.3]	0	Bet. (4 mg/day)	Until delivery	–	2	–	1 y	2
	Case 19		24	SS		16 4 [6.0]	0	Bet. (4 mg/day)	Until delivery	–	2	–	4 y	2
Sonesson et al. (43)	Case 20	PD MV-Ao +SVC-Ao	18–24?	–	Anti-SSA/Ro Anti-SSB/La	–	0	No therapy	Until delivery	–	3 (in 6 days when beta. started)	–	–	3
	Case 21–2	PD MV-Ao +SVC-Ao	18–24?	–	Anti-SSA/Ro Anti-SSB/La	–	–	No therapy	–	–	2	–	Few weeks	1
	Case 24–26					–	–	No therapy	–	–	1	–		1
Friedman et al. (37)	Case 27	PD MV-Ao	32	SLE/UAS/asym.?	Anti-SSA/Ro (Mean 16128)	170	–	No therapy	–	–	1	–	3 y	2
Skog et al. (44)	Case 28–30	Fetal echo	18–24?	SLE/SS/other?	Anti-Ro52	–	–	No therapy	–	–	2	–	1 m	1
	Case 31–35					–	–	No therapy	–	–	1	–		1
Jaeggi et al. (39)	Case 36	Fetal echo	22	-	Anti-SSA/Ro (55 U/ml)	–	–	No therapy	–	–	5	–	–	5
Izmirly et al. (40)	Case 37	–	–	SLE	Anti-SSA/Ro Anti-SSB/La	–	–	No therapy	–	–	2	–	3 y	2
Krishnan et al. (45)	Cases 38-41	PD MV-Ao	16–28?	–	Anti-SSA/Ro	>140?	–	No therapy	–	–	1	–	–	5
	Case 42										2	-	–	5
	Case 43		16–28?	–		160–170?	–	No therapy	–	–	2	–	–	5
Doti et al. (46)	Case 44	Fetal echo	25+	–	Anti-SSA/Ro Anti-SSB/La	–	–	No therapy	–	–	2	–	–	2
Cuneo et al. (42)	Case 45	PD MV-Ao	26+	–	Anti-SSA/Ro	–	–	No therapy	–	–	1	–	–	1
	Case 46		25+	–		–	–	No therapy	–	–	1	–	–	1
	Case 47		21+	–		–	–	No therapy	–	–	1	–	–	1

*GWs, gestational weeks; AVB, atrioventricular block; PD: pulsed-wave Doppler echocardiography; MV-Ao, from the intersection of the mitral E- and A-waves to the onset of the ventricular ejection wave in the aortic outflow; SVC-Ao, from the beginning of the retrograde venous a-wave in the SVC to the beginning of the aortic ejection wave; TDI, tissue Doppler imaging, echo: echocardiography; Aa-Sa, between atrial contraction and ventricular systole; FKCG, fetal kinetocariogram, SLE, systemic lupus erythematosus; UAS, undifferentisted autoimmune syndrome; asym., asymptomatic; SS, Sjögren syndrome; CTD, connective tissue disease; MCTD, mixed CTD; Dex., dexamethasone; Bet., betamethasone; Pre., prednisone; echo, echocardiogram; HCQ, hydroxychloroquine; IVIG, intravenous immune globulin; FGR, fetal growth restriction; LBW, low birth weight*.

*?: the specific value was not determine and only the range was provided in original study*.

*The bold type of word: highlighting the cases about the progression of the first-degree AVB to third-degree AVB*.

***Time from initiation to therapy**: 0 mean that the therapy for fetal first-degree AVB was started immediately after the diagnosis was made*.

***Prenatal results**: 1: conversation to sinus rhythm; 2: not progressed and sustained first-degree AVB; 3: progressed to third-degree AVB (complete AVB); 4: died; 5: alive but the detail conditions cannot be obtained from original study*.

***Follow-up results**: 1: healthy and normal development; 2: sustained first-degree AVB; 3: progressed to high-degree AVB; 4: died; 5: alive but the detail conditions cannot be obtained from original study probably attributed to loss of follow-up*.

It has been reported that in some studies untreated patients with first-degree AVB exhibited spontaneous recovery. We calculated a review of previous reports, 16/27 patients (59%) who did not receive treatment spontaneously converted to a normal sinus rhythm, which was maintained until delivery (Table 2). Overall the prenatal and follow-up ratios of conversion to sinus rhythm in the treated group were higher than in the untreated group (68 vs. 59% before birth, 72 vs. 63% during follow-up), but the differences were not statistically significant. Given that lower-degree AVB can progress to higher-degree AVB within 24 h, and that the adverse outcomes of third-degree AVB are irreversible, it is still recommended that the treatment of fetal first-degree AVB be considered with the agreement of guardians in cases where the mother and/or fetus exhibit life-threatening conditions. Transplacental dexamethasone (4–8 mg per day) and/or HCQ (200 mg two times per day) can be supplied for 4 weeks, and re-evaluation of the atrioventricular interval and fetal development is also required after treatment. A reduced dosage of dexamethasone should be administered after the first 2 weeks, and dexamethasone can be terminated if the atrioventricular interval decreases. If fetal development, as determined by the length of the femur and the biparietal diameter, has been identified as 2 weeks behind that of fetuses in the same GW, a more aggressive dexamethasone dosage reduction should be considered in an effort to maintain fetal growth.

**Table 2:** The comparison of fetal outcomes of autoimmune-associated first-degree AVB diagnosed initially between treated cases and untreated cases.

**Characterizes**	**Treated group (*n* = 20, 41.67%)**	**Untreated group (*n* = 28, 58.33%)**	***P*-value**
**Prenatal outcomes**			
Conversation to sinus rhythm	13 (65.00%)	16 (57.14%)	0.583
Persistence of first-degree AVB	6 (30.00%)	10 (35.71%)	0.679
Progression to second-/high-degree AVB	0	0	–
Progression to third-degree AVB	1 (5.00%)	1 (3.57)	1.000
Death *in utero*	0	0	–
Alive but unknown details	0	1 (3.57%)	–
Adverse effects on fetus	2 (10.00%)	–	–
**Postnatal outcomes**			
Sinus rhythm	14 (70.00%)	17 (60.71%)	0.507
First-degree AVB	4 (20.00%)	3 (10.71%)	0.429
Second-/high-degree AVB	0	0	–
Third-degree AVB	1 (5.00%)	1 (3.57%)	1.000
Death due to AVB or AVB therapy	0	0	–
Alive but unknown details in original study	1 (5.00%)	7 (25.00%)	–

*AVB, atrioventricular block*.

### Second-Degree AVB

Immune-associated second-degree AVB should be treated to avoid progression and adverse outcomes. Of the different degrees of AVB, treatment procedures for second-degree AVB have attracted the least debate. After a diagnosis of second-degree AVB has been made the therapeutic strategy involves oral administration of dexamethasone and HCQ, and intravenous immunoglobulin (IVIG). The IVIG should be administered four times at a dosage of 1 g/kg, within a period of 2 weeks. Additional administration should be continued once a month at a dosage of 1 g/kg if the initial treatment time is between the 16th and 30th GWs. The administration of dexamethasone and HCQ is the same as the strategy for first-degree AVB. Transplacental dexamethasone (4–8 mg per day) should be administered for 4 weeks, and HCQ (200 mg two times per day) should be considered for fetuses at all GWs. After 4 weeks of treatment echocardiography-based re-evaluation should be performed. If the atrioventricular interval is reduced, dexamethasone should be reduced or even terminated. The most important thing is to assess heart function. Second-degree AVB can lead to cardiac dysfunction. A β-sympathomimetic agent (terbutaline 2.5 mg every 8 h or salbutamol 2.4 mg every 8 h) can be used to increase heart rate, but there is currently insufficient evidence to support the administration of digoxin. Thus, the use of digoxin is an alternative that can be administered after due consideration. Preterm delivery of fetuses with normal heart function should be avoided.

### Third-Degree AVB

In fetuses with third-degree AVB the most important thing is to predict both fetal and maternal gestational outcomes. The avoidance of extremely adverse maternal effects should be afforded top priority. Lesions of the heart itself should be screened because the mortality rate is increased by > 50% in fetuses with endocardial fibroelastosis or dilated cardiomyopathy, and increased by nearly 100% when both lesions are present. Notably however, heart rate should be assessed first. If it is extremely low (55 bpm could work as a potential predictive value) the patient will die with severe heart dysfunction if there is accompanying endocardial fibroelastosis or dilated cardiomyopathy. In such cases pregnancy termination should be considered, to avoid adverse outcomes. Notably however, in some cases in which the heart rate has dropped below 55 bpm the patient has been kept alive by treatment with a combination of dexamethasone and β-agonists, with improved survival at 1 year and reduced morbidity (47). Such cases are critical though, and each needs to be carefully considered on an individual basis. However, the observational and therapeutic attempts are still considerable to under a totally agreement with mothers. Otherwise, immune-associated complete AVB should be treated via the same therapeutic strategy as second-degree AVB. Repeated echocardiography should be performed to facilitate detailed assessment of heart function and activity (34).

### Sinus Bradycardia

More and more studies found that the transplacental effects of anti-SSA/Ro and anti-SSB/La on fetal or neonatal cardiac rhythm are not only manifested as congenital atrioventricular block, but also manifested as sinus bradycardia. Chockalingam et al. observed that sinus bradycardia occurred after birth in neonates with positive maternal anti-SSA/Ro antibodies, and continued to exist in childhood (48). The treatment of sinus bradycardia without heart malformation is mainly observation and monitoring. Fetus with positive maternal anti-SSA/Ro and anti-SSB/La antibodies should be closely monitored for the changes of PR interphase to prevent the irreversible conduction bundle immune injury (34, 49, 50). For the symptoms of fetal bradycardia, pregnant women can use oral sympathetic adrenergic drugs to increase fetal heart rate and can use intravenous salbutamol followed by oral terbutaline maintenance treatment. Fetal heart rate can be increased by 15–25%. Isoproterenol has no significant effect on fetal heart rate (51, 52). Pacing therapy after birth is an option for children with slow heart rate. And since Carpenter had performed the first implantation of a fetal pacemaker (53), some researchers have been exploring fetal pacing therapy. For example a micro-pacemaker to treat severe fetal bradycardia is currently undergoing design which may become an option in the future (54).

### Controversies on Therapeutic Strategy

According to Diagnosis and Treatment of Fetal Cardiac Disease: A Scientific Statement from the American Heart Association (55), dexamethasone can prevent fetal cardiomyopathy or death from immune-associated second-degree and third-degree AVB with inflammatory symptoms. During treatment, fetal growth and development should be considered a key parameter when deciding whether to continue or terminate treatment. Depending on clinical follow-up observations, steroids can reverse the extension of the atrioventricular interval during treatment for first-degree AVB (38). Notably however, the risks may outweigh the benefits. Long-term administration of oral dexamethasone (56) is associated with increased body mass, hypertrichosis, mood changes, insomnia, hypertension, abnormal glucose tolerance, infection, cataracts, and bone mineral density problems in pregnant mothers, along with reduced amniotic fluid, growth restriction, and compromised neurodevelopment in the fetus. The administration of dexamethasone should thus be considered with caution, and treatment for first-degree AVB should be administered after due evaluation of the potential adverse effects and benefits of dexamethasone. The use of drugs should be reduced or even stopped in the event of conversion or failure to achieve therapeutic goals. The RRNL report demonstrated the failure of dexamethasone administration to prevent disease progression, reduce mortality, and avoid pacemaker implantation and cardiomyopathy in cases of second-degree and third-degree AVB (57). Similar results have been observed in a series of studies conducted by different research groups, challenging the therapeutic role of dexamethasone in fetal CHB management (6, 58). According to a systematic review and meta-analysis of maternal steroid therapy for fetuses with second-degree immune-mediated congenital AVB that was reported in 2018 (59), there is still limited evidence of the advantages of steroid administration with respect to fetal outcomes. A recent meta-analysis demonstrated fluorinated steroids were not superior to any treatment to ameliorate the outcome of autoimmune associated CHB (60).

β-agonists such as terbutaline and salbutamol have favorable transplacental transfer rates and β-agonist action, and they can increase fetal heart rate and mediate a positive inotropic effect on the fetal myocardium. They are usually administrated in combination with dexamethasone. According to the follow-up results of 37 cases of fetal complete AVB treated by Jaeggi et al. (47), hormone or sympathetic drug administration could improve the survival rate of fetal complete AVB, reduce the incidence of immune-related complications, and improve prognoses. Conversely however, Lopes et al. (61) reported the results of long-term treatment and follow-up of 116 fetal AVB patients, and in that cohort steroids and sympathetic drugs did not reverse fetal AVB or improve the survival rate.

**Figure 1:**
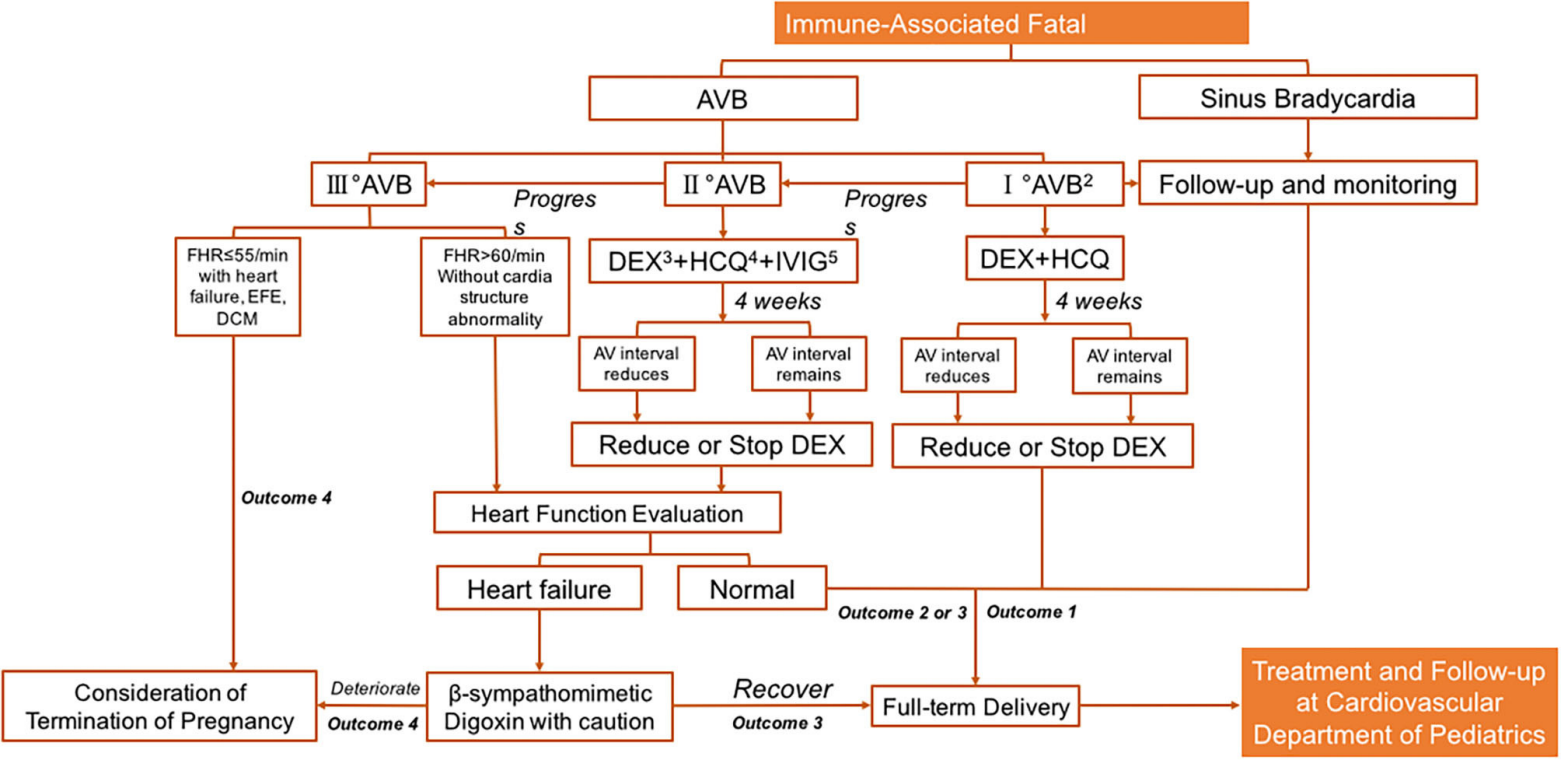
Prenatal management for immune-associated bradycardia. Note 1: Immune-associated: this figure refers specifically Anti-Ro/SSA (+) or Anti-La/SSB (+). Note 2: PR interval>150 ms. Note 3: Dexamethasone: 4–8 mg/qd, monitor the decrease of amniotic fluid, the length of fetal femur and the delay of fetal biparietal diameter by 2 weeks etc. Reduce and stop drugs in time. Note 4: Hydroxychloroquine: 200 mg po bid, continuous use during pregnancy. Outcome 1: I°AVB and sinus bradycardia do not require pacing therapy in childhood, and long-term follow-up is performed. Note 5: Intravenous immunoglobulin usage: (1) 1 g/Kg ivgtt, twice a week for 2 weeks, and then used according to the situation; (2) 1 g/kg ivgtt at 16–30 weeks of gestation, and 1 g/kg ivgtt once a month after 2 weeks of continuous use. Outcome 1: The sinus bradycardia and part of I°AVB patients do not need treatment during fetal stage, and continuous observation is required. Outcome 2: II°AVB: most patients do not need pacing therapy after birth. According to their growth and development and clinical symptoms, protective or therapeutic pacing therapy can be considered. Outcome 3: For II°AVB or III°AVB with symptomatic bradycardia, cardiac dysfunction or low cardiac output; Congenital III°AVB with wide QRS, ventricular ectopic or cardiac dysfunction; Congenital III°AVB with ventricular rate lower than 55 times/min; Asymptomatic congenital patients with III°AVB and not wide QRS and normal cardiac function, a protective pacemaker may be considered for treatment. Outcome 4: Death *in utero*; Or a referral to a general hospital that can provide rapid life support and intrapartum pacing therapy. AVB, atrioventricular block; FHR, fatal heart rate; AV interval, the time interval between the beginning of atrial systole and the beginning of ventricular systolic ejection; EFE, endocardial fibroelastosis; DCM, dilated cardiomyopathy.

IVIG can reduce transplacental autoantibody transfusion and increase the release of anti-inflammatory factors. It can therefore be used in combination with dexamethasone in the event of endocardial fibroelastosis or impaired cardiac systolic function (57, 62, 63), but there is no solid evidence on the optimal dosage or duration of IVIG administration. A flowchart summarizing the durations and dosages used in the most promising study reported by Trucco et al. (64) in which 80% of patients remained alive 2.9 years after birth without heart transplantation, as well as experiences from our own clinical practice, is shown in Figure 1.

The toll-like receptor blocker HCQ can reduce the risk of immune-associated fetal AVB progression, and in pregnant women who took HCQ the prevalence of fetal CHB was lower than it was in those who did not (65). Subsequent RRNL studies suggested a protective role of HCQ by way of reducing fetal CHB in subsequent babies by 64% (66). In one study HCQ was strongly associated with maintaining healthy babies in anti-Ro-positive mothers with systemic autoimmune diagnoses, including systemic lupus erythematosus and Sjögren's syndrome (67). Most recently, Izmirly et al. published an important research on the prevention role of HCQ in fetuses of anti-SSA/Ro positive mothers, which revealed HCQ reduced the recurrence of CHB below the historical rate by >50%, and this drug is recommended for the secondary prevention of fetal cardiac disease in anti-SSA/Ro-exposed pregnancies (68). In the absence of adverse events, HCQ administration could be recommended until delivery (66, 69, 70).

To date many therapeutic studies on fetal immune-associated CHB have been reported, and herein we have summarized the general treatments and assessments utilized in a flowchart of hierarchical management and monitoring regimens. Due to the low incidence of fetal immune-associated CHB it is difficult to conduct large-sample prospective randomized controlled trials, and there are many biases in retrospective studies. Therefore, the conclusions of clinical studies with large samples are sometimes inconsistent, and controversy over treatment strategies still exists. Large prospective studies are necessary to comprehensively evaluate the efficacy of therapy with different drugs and combinations thereof.

## Complications and Prognosis

The prognosis of fetal immune-associated bradycardia depends on the severity of the disease, the extent of its effects on cardiac function, and whether it is associated with cardiomyopathy and endocardial fibroelastosis. Generally, increased anti-Ro52 (≥ 50 U/mL), cardiac malformation, early onset (<20 GWs), advanced AVB, lower atrial rate ( ≤ 120/min), reduced ventricular rate (≤ 55/min) with heart dysfunction, edema, cardiomyopathy, or endocardial fibroelastosis are associated with unfavorable prognoses and increased mortality (39, 61, 63). To improve the overall survival rate and prognoses of perinatal children with immune-associated CHB, optimal and timely drug interventions are necessary during the fetal period. Pacemaker implantation and integrative medication management of newborns are also important, in order to save lives. In subsequent pregnancies in mothers of affected babies, the therapeutic strategy should involve integrated administrative coordination among obstetrics, ultrasound, and pediatric departments from the prenatal period to the postnatal period, and potentially preventive treatment.

## Perspective: Whether Transplacental Medication Administration Benefits the Fetus

In past decades, extensive studies have investigated autoimmune-associated fetal CHB, and the knowledge they have yielded has facilitated a better understanding of multiple aspects of the condition. Severe fetal AVB results in fetal death. In 40–50% of patients fetal CHB is associated with transplacental transfusion of maternal autoantibodies. Patients with immune-related CHB will potentially benefit from anti-immune treatment, but hierarchical fetal immune-associated CHB management strategies remain a subject of debate. The current review has discussed the accessible practical clinical approaches to fetal immune-associated CHB management based on the degree of AVB and fetal heart function. Previous guidelines on prenatal cardiovascular disease management are predominantly focused on the diagnosis of fetal cardiac malformation, fetal heart failure, and fetal arrhythmia, with little attention paid to the progression of clinical management and prenatal follow-up duration prior to reaching a decision with regard to delivery or termination.

To date no standardized therapy for first-degree AVB has been established, and maternal dexamethasone or HCQ administration usually cannot reverse it. Notably however, sometimes treatment can evidently prevent progression of first-degree AVB to AVB of a higher degree. So that, we could not strongly positive on aggressive suppling dexamethasone and HCQ to such patients as there is no available indicator to predict the progress of severity of fetal AVB, as the adverse effects from steroids administration are still calling attention. Above we have summarized the currently available data and suggested consideration of the administration of dexamethasone and HCQ in some circumstances, but all therapeutic interventions should be monitored very closely and evaluated within 4 weeks. Immune-related second-degree AVB should be treated to avoid progression and adverse outcomes, and patients with second-degree AVB are the most likely to derive advantages from transplacental medication therapy. Notably however, current evidence does not support the administration of anti-immune treatment to fetuses with third-degree AVB. Medication to maintain heart function is recommended. This review is an attempt to discuss the limitations of prenatal management strategies, and clearly present specific accessible hierarchical therapeutic and fetal monitoring protocols.

Currently, the pathogenesis of such heart conduction disorder is still unknown well, as only a few parts could be identified positive for auto-immune antibodies. And the treatment of dexamethasone, HCQ and IVIG is partially efficient. Echocardiography has been widely used to diagnose fetal arrhythmias, but it does not accurately reflect electrical activity, which leads to limitations with regard to indicators for predicting rapid progression of CHB. This limits the administrative decisions that can be made based on it, in fetuses with first-degree AVB. Moreover, fetuses cannot be fitted with pace-makers, so almost nothing can be done for those with third-degree AVB.

Based on our understanding, a large amount of research remains to be done and no definitive end-point is envisaged in the near future. Future research may mainly be focused on accurate diagnostic methods based on the electrical activity of the fetal cardiac conduction system. Early predictors to identify rapid progression of AVB and heart dysfunction remain elusive. Most importantly, our knowledge of postnatal development in such patients—particularly with regard to neuro-motor and cardiac function restoration as well as the maturation of cardiomyocytes—is very limited. Further research investigating such issues is urgently required. The physiological processes involved in maternal antibody transportation to the fetal environment also warrant further research, as do genetic risk factors that affect placental morphology and functional development.

The aforementioned future research directions have already been launched. Current treatments still require detailed validation via large-cohort observational studies and other types of evidence-based medical studies. Notably however, there is an ethical issue with respect to conducting randomized clinical trials involving such patients. Several groups have attempted to underline the outcomes on different reasons inducing fetal AVB, including the impacts on prognosis from fetal hydrops. But the etiological guiding management strategy is required. Functional magnetic resonance imaging has been used to investigate whether such patients are likely to suffer neurological disorders (symptomatic or asymptomatic). Recent studies indicate that placental barrier function is impaired under adverse maternal conditions, and the medication transplacental ratio mainly depends on transporters. Accordingly, it is important to identify the characteristics of placental transporters for dexamethasone, HCQ, and digoxin to release the precious personal therapeutic administration with maternal gestational complications (diabetes, hypertension, obesity, etc.).

In the near future, large cohort studies will provide more detailed information on hierarchical management strategies. Greater understanding of the molecular basis of pathogenesis will lead to the development and administration of more effective medications. Lastly, a better understanding of the role of the placenta in disease and drug transportation may facilitate more individualized treatment regimens based on specific fetal and maternal conditions.

## Conclusion

Fetal immune-associated CHB is a life-threatening condition. Anti-Ro/SSA and anti-La/SSB antibodies are currently considered to be the predominant antibodies that contribute to the most common causes of fetal immune-associated CHB. Fetal echocardiography is a comparatively reliable method for diagnosing immune-associated CHB prenatally. Recent studies partially support positive treatment approaches for first-degree and second-degree AVB. Transplacental administration of dexamethasone, IVIG, and HCQ evidently cannot reverse third-degree AVB, but benefit the fetal prognosis. Treatment to maintain heart function via digoxin and β-agonists can evidently benefit delivery outcomes, but such patients always require pacemaker implantation soon after birth. This review is the first to summarize clinically accessible strategies for the management of autoimmune-associated CHB in a hierarchical manner, focusing on controversies surrounding the benefits of transplacental medication administration according to degrees of AVB.

## Author Contributions

HL, CT, and LQ wrote the paper. CT, CW, and HL performed the collected the data. YH and KZ provided supervision and administration for this manuscript. CW and YL reviewed and approved the manuscript. All authors contributed to the article and approved the submitted version.

## Conflict of Interest

The authors declare that the research was conducted in the absence of any commercial or financial relationships that could be construed as a potential conflict of interest.
